# Increased large-scale inter-network connectivity in relation to impulsivity in Parkinson’s disease

**DOI:** 10.1038/s41598-020-68266-x

**Published:** 2020-07-10

**Authors:** Jinsoo Koh, Yoshiki Kaneoke, Tomohiro Donishi, Takuya Ishida, Mayumi Sakata, Yasuhiro Hiwatani, Yoshiaki Nakayama, Masaaki Yasui, Hiroshi Ishiguchi, Masaya Hironishi, Ken-ya Murata, Masaki Terada, Hidefumi Ito

**Affiliations:** 1 Department of Neurology, Wakayama Medical University, 811-1 Kimiidera, Wakayama, Wakayama Prefecture 641-8510 Japan; 2 Department of System Neurophysiology, Wakayama Medical University, 811-1 Kimiidera, Wakayama, Wakayama Prefecture 641-8510 Japan; 3 Wakayama-Minami Radiology Clinic, 870-2 Kimiidera, Wakayama, Wakayama Prefecture 641-0012 Japan

**Keywords:** Neuroscience, Neurology

## Abstract

Impulsivity is a neuropsychiatric feature of Parkinson’s disease (PD). We investigated the pathophysiology of impulsivity in PD using resting-state functional magnetic resonance imaging (rs-fMRI). We investigated 45 patients with idiopathic PD and 21 healthy controls. Based on Barratt Impulsiveness Scale (BIS-11) score, PD patients were classified as higher (PD-HI) or lower impulsivity (PD-LI). Functional connectivity (FC) between various large-scale brain networks were analysed using the CONN toolbox. FC between the right frontoparietal network (FPN) and medial visual network (MVN) was significantly higher in PD-HI patients than PD-LI patients (false discovery rate [FDR]-adjusted *p* = 0.0315). FC between the right FPN and MVN had a significant positive correlation with total BIS-11 score (FDR-adjusted *p* = 0.010) and the attentional impulsivity (FDR-adjusted *p* = 0.046) and non-planning impulsivity subscale scores (FDR-adjusted *p* = 0.018). On the other hand, motor impulsivity subscale score had a significant negative correlation with the FC between the default-mode and salience networks (right supramarginal gyrus, FDR-adjusted *p* = 0.018; anterior cingulate cortex, FDR-adjusted *p* = 0.027); this trend was observed in healthy controls. The attentional and non-planning impulsivity, regarded as ‘cognitive’ impulsivity, may be associated with dysfunction in integration of perceptual information and flexible cognitive control in PD.

## Introduction

The estimated number of individuals with Parkinson’s disease (PD) is increasing, with 6.1 million individuals affected by PD in 2016^1^. Patients with PD suffer from various non-motor symptoms, among which impulse control disorders (ICDs) are a common neuropsychiatric symptom. ICDs and related disorders include pathological gambling, hypersexuality, binge eating, compulsive shopping, punding, hobbyism and dopamine dysregulation syndrome. ICDs can cause unproductive, harmful and even illegal activity^2^. ICD risk factors include male gender, cigarette smoking history, younger age of disease onset, history of drug or alcohol abuse, novelty-seeking personality and higher dose or long-term use of dopamine agonists^2–4^. Previous brain imaging studies have consistently suggested that meso-cortico-limbic-striatal circuit dysfunction is associated with ICDs^5–7^. In addition, a hyper-dopaminergic state within the meso-limbic-cortical pathway has been found in PD patients with ICDs^7,8^. Furthermore, some studies have suggested that dopaminergic abnormalities may be neurobiological markers of vulnerability to the development of ICDs^9–12^. The association of *DAT1* and *OPRM1* with ICDs in PD suggests a genetic predisposition may play a role as well^13^.

Recently, trait impulsivity without apparent ICD in PD patients has been proposed in several studies^14–17^. High impulsivity (HI) is a precursor of ICDs and can also lead to severe ICDs^18,19^. While HI may be within the spectrum of ICDs, the underlying pathophysiological mechanism differs between trait impulsivity and ICDs^20^. For instance, ICD has been associated with increased connectivity between the left subthalamic nucleus and the left parietal operculum, whereas trait impulsivity has been associated with a weak connectivity between the left putamen and the right inferior temporal gyrus^21^. Other studies investigating cortical thickness, tractography, or functional MRI have also reported inconsistent results^5–7,19–22^. One of the causes is that impulsivity contains multifaceted constructs.

To define simply, impulsivity is the tendency to act prematurely without foresight. However, impulsivity is multifaceted like most other behaviours and involves various forms of response inhibition, reward evaluation, motivation and cognitive control^23^. To assess the multifaceted constructs of impulsivity, various methods have been developed, including Barratt Impulsiveness Scale 11th version (BIS-11), Stop Signal Reaction Time (SSRT), 5-choice serial reaction time task, Hayling test, go/no-go task and Iowa gambling task. Among these, BIS-11, which is the most commonly used self-reported questionnaire, can measure subdomains of attentional (Iat), motor (Im) and non-planning (Inp) impulsiveness. Among these, Iat and Inp subscales are considered ‘cognitive’ impulsivity, whereas motor impulsivity is the tendency to ‘act without thinking’. It has been pointed out that the HI of PD is caused by different pathophysiologies of motor impulsivity and cognitive impulsivity^24^, but few studies have investigated them individually.

To elucidate the pathophysiological background of impulsivity in PD, in vivo brain function should be evaluated because abnormal function does not necessarily coincide with pathological changes. Although there are many limitations to the in vivo assessment of human brain, remarkable progress has been made in resting-state functional magnetic resonance imaging (rs-fMRI), which has become more popular than conventional event-related fMRI. Metabolic changes in brain due to stimulation are < 5%, and almost all forms of brain dysfunction can be detected at a resting state^25,26^. Furthermore, rs-fMRI can reduce noise compared to event-related fMRI, particularly in elderly patients with PD characterised by bradykinesia or bradyphrenia. rs-fMRI is capable of creating highly reproducible maps of large-scale neural networks in the human brain^27,28^. Changes within these networks have been observed in many neuropsychiatric diseases^29^. Although several studies investigated impulsivity in PD using rs-fMRI, increased connectivity within the salience network (SN) and default-mode network (DMN) and decreased connectivity within the central executive network were associated with ICDs in PD^30^. On the other hand, altered frontoparietal network (FPN), which plays an important role in instantiating and flexibly modulating cognitive control^31,32^, has been suggested in the pathophysiology of attention-deficit/hyperactivity disorder^33^. Other studies on impulsive/compulsive disorders such as addiction and binge eating have also revealed alterations in large-scale networks^34,35^. Thus, changes in large-scale networks are a suggested cause of impulsivity.

Although basal ganglia is important in the regulation of impulsivity as well as ICDs, it is difficult to accurately separate the blood-oxygen-level-dependent (BOLD) signals of basal ganglia subregions, such as the core and shell of the nucleus accumbens, due to the limited spatial resolution of fMRI. Furthermore, the low-frequency oscillation of BOLD signals has been shown to be slower in basal ganglia than in cerebral cortex^36^. Therefore, functional connectivity (FC) between basal ganglia and cortex can be underestimated during comparison with inter-cortex connectivity. On the other hand, subcortical regions regulate large-scale networks. For instance, the SN is affected by striatal disruption and SN dysfunction impacts the modulation of DMN^37^. Thus, disorders affecting subcortical regions are reflected in major large-scale networks. Therefore, we hypothesized that measuring the FC of large-scale networks of the cerebral cortex is an effective approach for evaluating complex psycho-cognitive functions such as impulsivity.

To test our hypothesis that the impulsivity in PD is related to alterations in large-scale networks such as the DMN, SN and FPN, we examined FC changes in various large-scale networks between PD patients grouped according to impulsivity using fully automated whole-brain FC analysis. For association with the basal ganglia, the FC of nucleus accumbens with closely related brain regions was measured separately based on the abovementioned issues. The present study, albeit exploratory, provides evidence that the change in the FC of large-scale networks is associated with the pathophysiology of impulsivity in PD.

## Results

### Patient characteristics

After enrolment, during the two-year follow-up period, the PD diagnosis of three patients changed: one to progressive supranuclear palsy, one to multiple system atrophy and one to psychogenic tremor; all three were excluded from analysis. Another patient was excluded because of new onset myoclonic epilepsy. We also excluded 20 patients with a mini-mental state examination (MMSE) score < 27 and three patients with < 250 valid imaging scans, for the accurate detection of FC abnormalities related to impulsivity in PD. Finally, 45 PD patients and 21 healthy controls (HCs) were included for analysis (Table 1).

**Table 1:** Patient characteristics.

	PD-HI N = 20	PD-LI N = 25	Control N = 21	*P* value
Gender, F/M	12/8	15/10	8/13	0.27^a^
Age, years	63.9 ± 10.2	65.8 ± 8.5	64.3 ± 10.3	0.78^b^
Education, years	12 (12–15.5)	12 (12–14)	12 (12–15)	0.88^c^
BIS-11 score	66.0 ± 4.3 65 (62.3–69)	54.4 ± 5.1 56 (50–58.5)	56.6 ± 10.6 57 (47–67)	< 0.0001^c^
Iat	17.1 ± 2.4	13.8 ± 1.8	14.0 ± 4.0	0.0001^b^
Im	22.2 ± 2.7	18.6 ± 3.0	19.8 ± 4.2	0.0031^b^
Inp	26.8 ± 3.1	22.0 ± 3.2	22.8 ± 5.4	0.0005^b^
Age at onset, years	56.8 ± 8.6	58.8 ± 2.1	–	0.47^d^
Duration, years	6.3 (2.8 − 10.3)	6.3 (2.4–9.0)	–	0.89^e^
LEDD, mg	463 (356–623)	475 (319–663)	–	0.97^e^
Levodopa, mg	300 (38–438)	300 (300–350)	–	0.85^e^
Dopamine agonist, mg/LEDD	150 (0–285)	150 (19–233)	–	0.73^e^
MMSE score	29.5 (28.25–30)	30 (29–30)	NA	0.38^e^
FAB score	15.5 (13–17)	16 (14–17)	NA	0.52^e^
Stroop	8 (4.5–13.8)	10 (5.5–16.5)	NA	0.37^c^
MDS-UPDRS score	47.6 ± 15.2	43.6 ± 25.7	NA	0.55^d^
Part 1	10 (6.25–13)	7 (5.5–9.5)	NA	0.047^e^
Part 2	12.5 (9.3–16.8)	9 (4–12.5)	NA	0.044^e^
Part 3	22.2 ± 7.7	23.8 ± 15.9	0	0.69^d^
Part 4	0 (0–2.75)	1 (0–5)	NA	0.17^e^

Continuous variables are presented as means ± standard deviation and/or medians (interquartile range), depending on data distribution.

*BIS-11* Barratt impulsiveness scale 11th version, *LEDD* levodopa-equivalent daily dose, *MMSE* mini-mental state examination, *FAB* frontal assessment battery, *MDS-UPDRS* movement disorder society-sponsored unified Parkinson’s disease-rating scale, *NA* not available.

^a^Fisher’s exact test.

^b^One-way analysis of variance.

^c^Kruskal–Wallis test.

^d^*t*-test between PD-HI and PD-LI.

^e^Wilcoxon rank-sum test between PD-HI and PD-LI.

There were no significant differences between the PD patients and HCs with respect to age, gender and education. The median BIS-11 score was 60 in the PD patients; those with BIS-11 score ≥ 60 were classified as PD-HI. The mean BIS-11 score in the PD-HI group was similar to that reported in PD patients with ICDs^4^. Median BIS-11 score was 57 in the HCs. There was no significant difference in Im between the PD-HI and HC groups; however, there were significant differences in other BIS-11 total and subscale scores between the PD-HI group and the other groups. The total BIS-11 score and each subscale score did not significantly differ between the PD-LI patients and HCs.

Although not significant, PD-HI patients tended to be slightly younger than PD-LI patients, and the total levodopa-equivalent daily dose (LEDD) tended to be lower in PD-HI patients. MMSE score, frontal assessment battery (FAB) score, Stroop test and Movement Disorder Society-Sponsored Revision of the Unified Parkinson’s Disease-Rating Scale (MDS-UPDRS) part 3 and part 4 scores were not significantly different. MDS-UPDRS part 1 and part 2 scores were significantly higher in the PD-HI patients. The part 1A subscale scores did not significantly differ between the PD-HI and PD-LI patients, including #1.2 hallucination/psychosis (*p* = 0.18) and #1.6 dopamine dysregulation syndrome (*p* = 0.19). Multiple regression analysis did not show any significant association between BIS-11 score and other clinical parameters.

### Whole-brain functional connectivity analysis of large-scale networks

**Figure 1:**
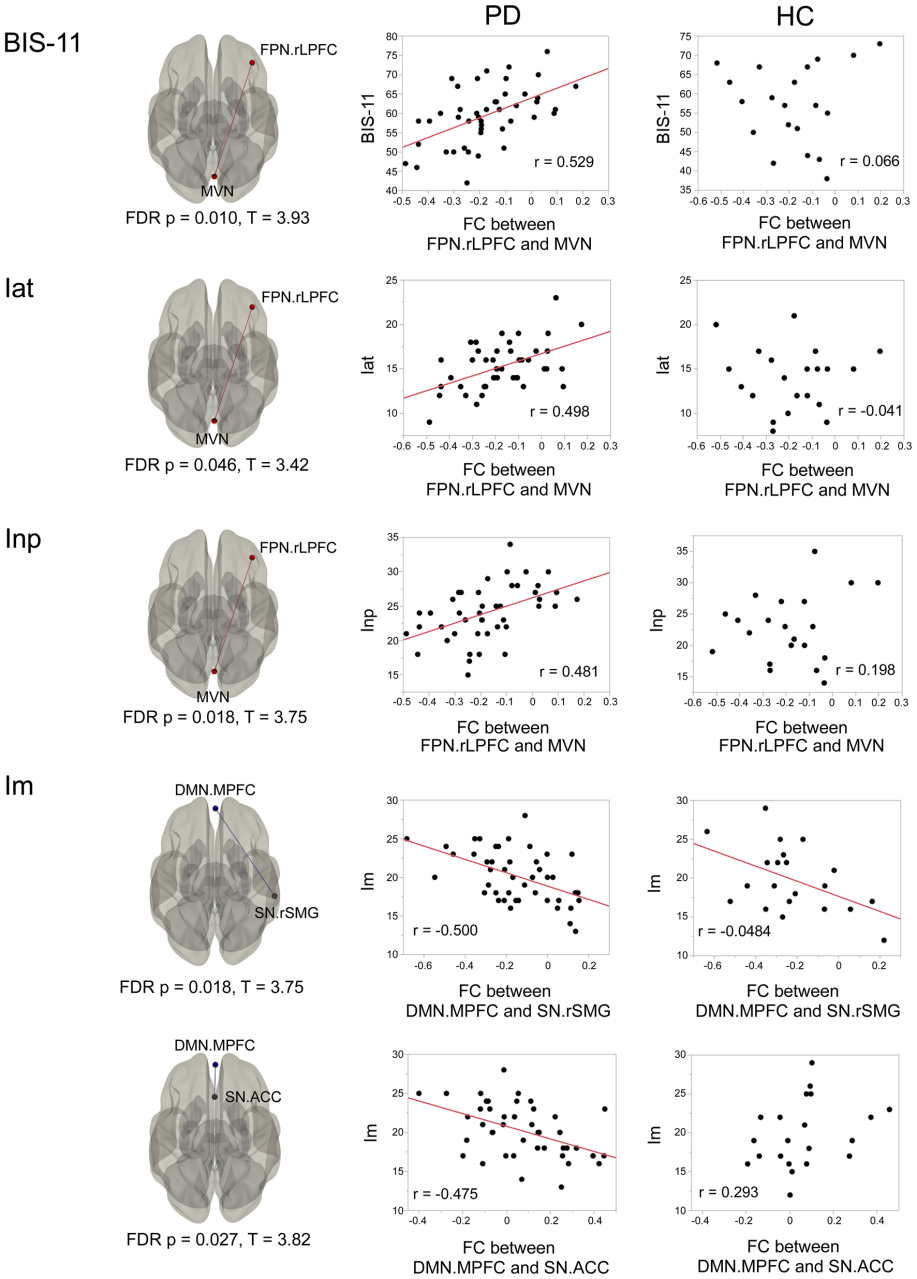
Comparison between BIS-11 score and inter-network functional connectivity (FC). Bivariate correlation analysis shows significant positive correlation between FC of FPN-MVN and total BIS-11 and Iat and Inp subscale scores. Higher Im subscale score is related to decreased FC between the DMN and salience networks. Regarding healthy controls, the FC between DMN and right SMG of SN tended to be negatively correlated with Im subscale score. *rLPFC* right lateral prefrontal cortex, *FPN* frontoparietal network, *MVN* medial visual network, *ACC* anterior cingulate cortex, *rSMG* right supramarginal gyrus, *DMN* default-mode network.

FC was significantly higher between the right lateral prefrontal cortex (LPFC) of the FPN and the medial visual network (MVN) in PD-HI patients than PD-LI patients (false discovery rate [FDR]-adjusted *p* = 0.0315, T = 3.55). Figure 1 illustrates the results of the correlation analyses between FC and the total BIS-11 and subscale scores. The total BIS-11 score and Iat and Inp subscale scores in PD patients showed a significant positive correlation with FC between the right LPFC and MVN. This finding persisted even when the MDS-UPDRS part 3 score was included as a nuisance covariate. Only the Im subscale score showed a different tendency: higher score was related to decreased FC between the DMN (medial prefrontal cortex) and SN (supramarginal gyrus and anterior cingulate cortex). Regarding the HCs, the FC between large-scale networks was not significantly correlated with the BIS-11 score or the subscale scores. However, the FC between DMN and right supramarginal gyrus, which was significantly correlated with the Im score in patients with PD, tended to be negatively correlated with the Im score in HCs (Fig. 1).

### Functional connectivity analysis of basal ganglia

There were no significant differences in the FC between basal ganglia and large-scale networks between the PD-HI and PD-LI groups. There were also no significant differences in the FC between nucleus accumbens and other closely related regions among the three groups (Table 2).

**Table 2:** Functional connectivity between nucleus accumbens and other regions.

	PD-HI	PD-LI	Control	*p*
**R Accumbens**				
ACC	0.081 ± 0.133	0.056 ± 0.102	0.063 ± 0.118	0.77
R Putamen	0.094 ± 0.118	0.065 ± 0.117	0.096 ± 0.108	0.59
R Amygdala	0.061 ± 0.130	− 0.010 ± 0.112	0.034 ± 0.098	0.12
**L Accumbens**				
ACC	0.157 ± 0.150	0.104 ± 0.090	0.123 ± 0.099	0.31
L Putamen	0.131 ± 0.088	0.109 ± 0.107	0.132 ± 0.121	0.70
L Amygdala	0.085 ± 0.102	0.060 ± 0.115	0.036 ± 0.109	0.36

Continuous variables are presented as means ± standard deviation. Comparisons among the three groups were determined by analysis of variance.

*R* right, *L* left, *ACC* anterior cingulate cortex.

### Voxel-based morphometry results

Grey matter (GM) volume in the left inferior temporal cortex was significantly different among the three groups (Montreal Neurological Institute (MNI) xyz coordinates: − 54, − 12, − 29); the volume was significantly lower in the PD-HI patients compared to HCs. Multiple regression analysis detected no significant correlation between GM volume in the left inferior temporal cortex and BIS-11 score in patients with PD.

## Discussion

In the present study, we showed FC differences among large-scale networks in relation to trait impulsivity of PD. While the BIS-11 total score and the Iat and Inp subscale scores were related to increased FC between the right FPN and MVN in PD patients, the Im subscale score was related to decreased FC between the DMN and SN. Since Iat and Inp are regarded as ‘cognitive’ impulsivity^24^, this study could successfully detect the difference in the pathophysiology between cognitive and motor impulsivity in PD. Although no study to date reported the association between ‘cognitive’ impulsivity and right FPN, the FPN plays an important role in instantiating and flexibly modulating cognitive control^25,26^. Therefore, our findings suggest that abnormal cognitive control due to dysfunction of FPN is related to ‘cognitive’ impulsivity in PD.

Several studies investigated changes in the FC of DMN and SN related to impulsivity^38,39^. In particular, a decrease in the FC of DMN has been identified in pathological gambling^40^, and the association between motor impulsivity and pathological gambling has been demonstrated in a meta-analysis^41^. Thus, the abnormalities in FC between the DMN and SN in relation to motor impulsivity were reproducible in the present study. Further, a similar tendency in the FC between the DMN and right supramarginal gyrus of SN was observed in HCs. Therefore, this finding may reflect the characteristics of individual ‘model-based’ learning rather than PD-specific changes.

The involvement of the MVN in HI remains unclear. Ide et al. also unexpectedly found that increased GM volume in the bilateral medial parietal and occipital cortices in healthy adults was associated with total BIS-11 score and Iat and Inp subscale scores^42^. Although they could not specify the pathophysiology, they speculated that vulnerability to distraction by visual stimuli was involved. The ventral pathway of visual information is integrated in the inferior temporal gyrus with top down control from the prefrontal cortex. Therefore, our results, albeit not conclusive, may reflect dysfunction in the integration of visual information. Alternatively, the amount of visual information might be superior to other perceptual information or the variance in BOLD signals might have been higher in the occipital lobe than in the other regions^43^. On the other hand, visual network alterations are also associated with visuospatial and visual hallucinations in PD^44^. In the present study, few patients experienced visual hallucinations, and there was no significant difference in the hallucination score between the PD-HI and PD-LI groups. We could not exclude the potential contribution of slight visuospatial impairment because we did not conduct optimal visuospatial assessment; however, none of the patients had difficulty in drawing pentagons during the MMSE. In addition, a relatively strict cut-off MMSE score of > 27 was adopted to minimise the effects of slight visuospatial cognitive impairment.

The association of impulsivity with cognitive dysfunction in PD remains controversial. Although some reports have suggested an association between frontal lobe dysfunction and impulsivity^45^, others have suggested that impulsivity is independent of the cognitive state^46,47^. The multifaceted nature of impulsivity may explain these contradictory results. In the present study, MMSE, FAB and Stroop test did not differ between the PD-HI and PD-LI groups, which may be due to the relatively stringent criteria for MCI exclusion and the small number of cases.

Previous reports clearly show that basal ganglia play an important role in regulating impulsivity. However, the present study could not detect any association between the impulsivity and FC of basal ganglia. This negative finding may be explained by false-negative data associated with the abovementioned limitations of rs-fMRI or may indicate that the inter-cortical connectivity may be more important than basal ganglia for impulsivity in PD in the absence of ICDs. However, it is unlikely that basal ganglia are not involved in impulsivity control at all. Therefore, these findings should be considered to be associated with changes in the regulation of cortex via basal ganglia. In other words, it is highly possible that the failure to detect a direct FC abnormality in basal ganglia is a false-negative finding because of the methodology and that the FC abnormality between the cortices is regulated by basal ganglia. Future studies by other methodologies are necessary to further explore this possibility.

Although we found left inferior temporal atrophy in HI patients, consistent with one previous study^5^, another study did not^48^. Although this might be a false-positive finding, our rs-fMRI results were not influenced by inferior temporal atrophy, because the inferior temporal lobe is not included in the large-scale network regions of interest of the CONN toolbox.

In addition to these caveats, the present study has several limitations. First, MRI examinations were performed with the patient in the ‘on’ state while taking their usual medication to avoid the burden of dopamine discontinuation and reduce motion artefact, as previously reported^6,49^. Therefore, we cannot exclude the direct effect of dopamine replacement therapy (DRT) on FC. However, it is not feasible to eliminate the effects of DRT on FC. Although levodopa modifies FC in the short-term[^50^], there is no evidence of long-term influence. Thus, we attribute the current findings to changes of FC under usual conditions, considering the effect of DRT. Second, we used BIS-11 as the sole measure of impulsivity and did not include other evaluation methods such as SSRT. In the present study, we focused on examining rs-fMRI in a relatively large number of cases and excluded other methods due to feasibility concerns. The QUIP-RS, an ICD questionnaire for PD that is also used to assess compulsivity in PD^51^, has not yet been validated in Japanese. A more accurate understanding of the pathophysiology underlying impulsivity requires comparison of BIS-11 with other methodologies.

In conclusion, we have demonstrated that ‘cognitive’ impulsivity in PD is associated with increased large-scale inter-network FC changes between the right FPN and MVN. This new finding suggests that ‘cognitive’ impulsivity in PD may be associated with dysfunction of flexible cognitive control via the FPN. Although the involvement of the MVN is unclear, the integration of visual information might be involved. Our analyses also suggest an association between motor impulsivity and altered FC in the DMN and SN in both PD patients and HCs. Further studies are necessary to elucidate the pathophysiology of impulsivity in PD.

## Methods

### Participants

We recruited 72 consecutive PD patients and 21 HCs. All patients with PD fulfilled the clinical diagnostic criteria of the United Kingdom Parkinson’s Disease Society Brain Bank. Patients with apparent dementia and those receiving any cholinesterase inhibitor or antipsychotic medication were excluded from initial enrolment. The HCs were either partners of patients with PD or volunteers without PD as assessed by MDS-UPDRS part 3. We also excluded participants with ICDs based on assessment by a semi-structured interview as previously reported^52^: we used the diagnostic statistical manual of mental disorders IV-TR criteria for pathological gambling and binge eating^53^ and the currently proposed research criteria for hypersexuality, excessive buying^54,55^, punding, hobbyism and dopamine dysregulation syndrome[^55^^–^^57^], diagnosed by a board-certified neurologist (J. K.).

This study was conducted according to the principles of the Declaration of Helsinki and approved by the Wakayama Medical University Ethics Committee. All participants provided written informed consent.

### Assessment of impulsivity and clinical symptoms

To assess impulsivity, we applied the Japanese version of the BIS-11[^58^], which is the most commonly used instrument designed to assess impulsivity^59^. It has also been used to assess impulsivity in patients with PD^14,15^. The BIS-11 contains three subscales: Iat, Im and Inp. Each patient with PD was classified as PD-HI or PD-LI, based on the median BIS-11 score (PD-HI, BIS-11 score ≥ 60; PD-LI, BIS-11 score < 60). For motor and neuropsychiatric assessment of patients with PD, we used the MDS-UPDRS, MMSE, FAB and Stroop test during the ‘on’ state, assessed by board-certified neurologists. The LEDD was calculated as previously reported^60^.

## 3 MRI acquisition

A 3-T MRI scanner with a 32-channel head coil was used to scan each participant. To minimise motion artefact, the patients underwent MRI during the ‘on’ state while taking their usual medications, as in previous studies^6,7,61^. The following parameters were used to acquire T1-weighted structural images: TR = 7 ms, TE = 3.3 ms, field of view (FOV) = 220 mm, matrix scan = 256, slice thickness = 0.9 mm and flip angle = 10°. The following parameters were used for functional data using a gradient-echo echo-planar pulse sequence sensitive to BOLD contrast: TR = 3000 ms, TE = 30 ms, FOV = 192 mm, matrix scan = 64, slice thickness = 3 mm, number of slices 38 and flip angle = 80°, which covered the entire cerebral GM and cerebellum. Three runs, each with 105 volumes, were performed for each participant. In total, data were acquired over approximately 15 min during a resting state for each participant, as this duration was deemed the most appropriate to obtain reliable data^26,62^. The participants were asked to remain awake but keep their eyes closed during the acquisition.

### Functional MRI data preprocessing

We used Statistical Parametric Mapping software, version 12 (SPM12) and CONN toolbox, version 17 in Matlab R2014a for MRI data preprocessing. The CONN toolbox is designed to preprocess both functional and anatomical files, including realignment, slice-timing correction, outlier identification, co-registration, segmentation, normalisation and smoothing^63^. Preprocessing of functional data was performed by the default preprocessing pipeline. The initial five scans were removed to eliminate equilibration effects. Images were realigned and unwarped. Slice-timing was corrected in ascending order. For functional outlier detection, we used intermediate settings (97th percentile in normative sample), by adaptive resonance theory implemented in CONN. With a series of three runs of functional imaging acquired, we analysed a total of 300 functional images per subject. For the accuracy of the analysis, we included only subjects who had more than 250 valid scans in the final analysis. Both functional data and structural data were segmented to GM, white matter (WM) and cerebrospinal fluid (CSF) and normalised to MNI coordinates. Functional images were smoothed to 8 mm full width at half maximum (FWHM) Gaussian kernel. Subsequently, denoising was performed by default, including band-pass filtering (0.008–0.09 Hz) and linear regression.

### Whole-brain functional connectivity analysis of large-scale networks

For the first-level analysis between regions of interest (ROIs), we used the 32 ROIs of large-scale networks from the CONN toolbox as defined by its independent component analyses of the Human Connectome Project dataset (497 subjects): default mode (4 ROIs), sensorimotor (3 ROIs), visual (4 ROIs), salience/cingulo-opercular (7 ROIs), dorsal attention (4 ROIs), frontoparietal/central executive (4 ROIs), language (4 ROIs) and cerebellar (2 ROIs). For the second-level analysis, the two-sample *t*-test was applied to evaluate the difference between PD-HI and PD-LI patients (nuisance covariates: age, gender, LEDD; with and without MDS-UPDRS part 3 scores). Bivariate correlation analysis was performed to evaluate the effect of total BIS-11 and subscale scores. All effects were evaluated using an FDR-adjusted p value of 0.05.

### Functional connectivity analysis of basal ganglia

For the first-level analysis of FC of basal ganglia, we used ‘atlas’ ROIs from the CONN toolbox. For the second-level analysis, the two-sample t-test was used to evaluate the difference between PD-HI and PD-LI patients (nuisance covariates: age, gender and LEDD).

Each FC value (Z score of correlation coefficient) between the nucleus accumbens and other regions (such as putamen, amygdala and ACC) was also calculated in PD-HI, PD-LI and HCs.

### Structural MRI data analysis

For voxel-based morphometry, we used SPM12 in Matlab. T1-weighted structural images were segmented to GM, WM, CSF and other non-brain portions. Spatial normalisation was performed on GM probability maps using the diffeomorphic anatomical registration with exponentiated Lie algebra algorithm. Normalised imaging was smoothed using 8 mm FWHM Gaussian kernel. Age and gender were included as nuisance covariates. We applied total brain volume for global calculation. Both implicit and explicit masking were used. To evaluate differences between the PD-HI and PD-LI patients and HCs, we performed one-way analysis of variance (ANOVA) and the two-sample t-test. Tests were evaluated using family-wise error correction. *p* < 0.05 was considered significant for ANOVA; *p* < 0.05/3 was considered significant for the two-sample *t*-test.

### Statistical analyses for patient characteristics

Patient characteristics were analysed using JMP Pro14 software. Normality of data was assessed using the Shapiro–Wilk test. Categorical variables are presented as numerals. Normally distributed continuous variables are presented as means and standard deviation. Non-normally distributed continuous variables are presented as medians and interquartile range. The PD-HI and PD-LI patients and HCs were compared using ANOVA with post-hoc Tukey-HSD test or the Kruskal–Wallis test; two-group comparisons were performed using the unpaired t-test or Wilcoxon rank-sum test. Categorical variables were compared using Fisher’s exact test. Multiple regression analysis was performed to examine association with BIS-11 score. *p* < 0.05 was considered significant.

## Data Availability

The datasets analysed during the current study are available from the corresponding author on reasonable request.
